# Polyurethane Foams for Thermal Insulation Uses Produced from Castor Oil and Crude Glycerol Biopolyols

**DOI:** 10.3390/molecules22071091

**Published:** 2017-07-02

**Authors:** Camila S. Carriço, Thaís Fraga, Vagner E. Carvalho, Vânya M. D. Pasa

**Affiliations:** 1 Laboratório de Produtos da Biomassa, Departamento de Química, Universidade Federal de Minas Gerais, Av. Antonio Carlos, 6627, 31270-901 Belo Horizonte, Minas Gerais, Brazil; cscarrico@ufmg.br (C.S.C.); tf.thais@hotmail.com (T.F.); 2 Laboratório de Física de Superfícies, Departamento de Física, Universidade Federal de Minas Gerais, Av. Antonio Carlos, 6627, 31270-901 Belo Horizonte, Minas Gerais, Brazil; vagner@fisica.ufmg.br

**Keywords:** polyurethane foams, castor oil, crude glycerol, biopolyols, thermal insulator

## Abstract

Rigid polyurethane foams were synthesized using a renewable polyol from the simple physical mixture of castor oil and crude glycerol. The effect of the catalyst (DBTDL) content and blowing agents in the foams’ properties were evaluated. The use of physical blowing agents (cyclopentane and n-pentane) allowed foams with smaller cells to be obtained in comparison with the foams produced with a chemical blowing agent (water). The increase of the water content caused a decrease in density, thermal conductivity, compressive strength, and Young’s modulus, which indicates that the increment of CO_2_ production contributes to the formation of larger cells. Higher amounts of catalyst in the foam formulations caused a slight density decrease and a small increase of thermal conductivity, compressive strength, and Young’s modulus values. These green foams presented properties that indicate a great potential to be used as thermal insulation: density (23–41 kg·m^−3^), thermal conductivity (0.0128–0.0207 W·m^−1^·K^−1^), compressive strength (45–188 kPa), and Young’s modulus (3–28 kPa). These biofoams are also environmentally friendly polymers and can aggregate revenue to the biodiesel industry, contributing to a reduction in fuel prices.

## 1. Introduction

Rigid polyurethane foams are usually applied as thermal insulation in buildings, as well as in automobile and aerospace industries. The global rigid polyurethane foam market is expected to reach USD $20.40 billion by 2020 in construction applications, such as in residential and commercial roofs, walls, panels, and doors, and in appliance applications [1]. For insulation purposes, thermal conductivity is the most important property to be studied. Typical thermal conductivity values for polyurethane foams are between 0.02 and 0.03 W·m^−1^·K^−1^ and the very efficient thermal insulation of these materials is due to the extremely low thermal conductivity of the blowing agent gas trapped in the closed porous structures [2,3]. Additives, as blowing agents, catalysts, and surfactants, are very important to adjust the final properties of the foam’s synthesis from the main reaction of an isocyanate with a polyol. However, most of these reagents are derived from petrochemicals, increasing the petroleum dependence and, as a consequence, environmental problems. These aspects have encouraged the production of rigid foams from renewable materials [4].

In recent years, the uses of bio-based polyols have been investigated to produce sustainable and eco-friendly rigid polyurethane foams, such as lignin [5,6], biopitch [7,8], glycerol [9,10,11], and vegetable oils including castor [3,12,13,14,15,16,17,18,19], palm [20,21], soybean [11,22] starch [23,24], etc. Castor oil (CO) is a mixture of triglycerides, mainly ricinoleic acid, which is produced from the seed of the *Ricinus communis* plant. This low-cost renewable raw material has been usually used as a polyol for flexible material production, as polyurethane foams and elastomers. However, most of these materials are produced after chemical modifications of castor oil, such as transesterification, oxypropilation, hydroformilation, and ozonolysis, in order to introduce reactive hydroxyl groups, which involve oil pre-treatment with multiple steps and high costs.

Castor oil has high molecular weight, a low hydroxyl number, and poor reactivity, and also a slow cure time, low flame retardancy, and low miscibility with other components. These properties have limited the use of castor oil as a raw material polyol, especially to prepare rigid polyurethane foams, and the pre-treatment reaction seems to be efficient to minimize these disadvantages. Transesterification [16,18,19,25], thio-ene [26], and amidization [27] reactions have been employed to introduce reactive OH groups in castor oil in order to produce suitable polyols to synthesize rigid polyurethane foams. Recently, a work was published presenting a polyol production from the polymerization of glycerol followed by condensation of this polymerized material with castor oil. After this, rigid polyurethane foams were synthesized by partial substitution of a petrochemical polyol with this produced polyol [28]. These reactions are usually performed in the presence of an alcohol, and the use of glycerol has also been extensively studied. These foams presented good mechanical properties: densities of 35–50 kg·m^−3^, compressive strengths of 127–475 kPa, and also great thermal stabilities, with a limit oxygen index of 20–30%, and a thermal conductivity of 0.021–0.029 W·mK^−1^. However, the reactions to modify the oil and enhance its reactivity are conducted between 160 to 240 °C, for 2–6 h, involving multiple steps and some reactants, which increases the foam costs.

In the present study, polyurethane foams were prepared using a simple mixture of crude glycerol and pure castor oil. Binary mixtures with different contents of castor oil and crude glycerol were prepared in order to increase the functionality of this biopolyol without the extra costs of chemical modifications. The mixtures were achieved because the crude glycerol has three hydroxyl groups and a short chain, as well as good miscibility in the castor oil. The polyol was produced from the physical mixing of raw materials without any type of pre-treatment, and for direct use in polyurethane synthesis. This simple and innovative approach allowed for the production of renewable rigid polyurethane foams with good properties for thermal insulation uses. The effects of the catalyst and blowing agent in the mechanical and thermal conductivity properties of the foams were evaluated. This process is inexpensive, simple, and sustainable, and suitable to produce environmentally friendly materials with commercial applications.

## 2. Materials and Methods

### 2.1. Materials

Castor oil used was provided by PolyUrethane Company (Betim, Minas Gerais, Brazil). Crude glycerol, a co-product of biodiesel production, was kindly provided by Petrobrás (Usina Darcy Ribeiro—Montes Claros, Minas Gerais, Brazil). The crude glycerol has about 91% of glycerin, 5% of inorganic compounds (transesterification catalyst, acids, etc.), 1% of methanol, and about 3% of water and biodiesel, oleine, monoglicerides, and others. Pure glycerol (99.5%) was supplied by Synth. The isocyanate source (Desmodur 44 V 20), a mixture of 4,4′-diphenylmethane-diisocyanate, was supplied by Bayer Company. The surfactant was Tegostab 8460 (Evonik, Essen, Germany), a polyether-modified polysiloxane. The catalyst used in polyurethane foams synthesis was DBTDL (dibutyl tin dilaurate), an organometallic catalyst, manufactured by Evonik. The blowing agents used were cyclopentane (Sigma Aldrich, St. Louis, MO, USA), n-pentane (Sigma Aldrich, St. Louis, MO, USA), and distilled water.

### 2.2. Polyol Preparation

Polyols were obtained by binary mixtures of pure glycerol (P.A. glycerol) and castor oil in different proportions (20%, 40%, 50%, 60% and 80% of pure glycerol *w*/*w*). Biopolyols were also prepared from binary mixtures with different amounts of castor oil (Co) and crude glycerol (G), called GCo (10%, 20%, 30%, 40%, 50%, 60% and 70% of crude glycerol *w*/*w*). The raw materials were added in a beaker and homogenized for 1 min using a mechanical stirrer (Fisatom model 713 D, São Paulo, SP, Brazil).

### 2.3. Foam Synthesis

#### 2.3.1. Study of the Best Binary Mixture to Produce Polyurethane Foams

A preliminary study to choose the best polyol formulation was carried out. For this purpose, rigid polyurethane foams were synthesized using the batch process method and binary polyols with different compositions. This comparative study was performed with the mixture of P.A. glycerol (20%, 40%, 50%, 60%, 80% *w*/*w*) and castor oil, and with crude glycerol (10%, 20%, 30%, 40%, 50%, 60%, 70%) and also castor oil. The NCO/OH molar ratio was kept equal to 2.0, the surfactant, 2% (*w*/*w*), and water was employed as the blowing agent (2%). These mixtures were kept under vigorous stirring for 1 min at 500 rpm. Afterwards, the isocyanate was added to the pre-mixture and stirred until complete homogenization. The formulations were poured into a wooden mold with the dimensions 7.0 × 7.0 × 20.0 cm for the growth of the polymer foams and cured for 24 h at room temperature. After the curing time, the foams were demolded and visual inspections were performed. The best polyol formulation was chosen for the foam with the best properties (aspect, homogeneity, dimensional stability, and low friability).

#### 2.3.2. Study of Catalyst and Blowing Agent Effect on the Foams Properties

After preliminary tests, the best binary polyol GCo with 10% crude glycerol, and 90% castor oil *w*/*w* was used to produce foams with good thermal stability, mechanical properties, and low thermal conductivity for insulation use. For this purpose, this best polyol was tested to prepare foams aiming to evaluate the effect of different catalyst (DBTDL) amounts (1% and 2% *w*/*w*); different blowing agent types (water, n-pentane, and cyclopentane), and contents. The tests to evaluate the different blowing agent amounts were carried out with: water = 2%, 4% and 6% *w*/*w*; n-pentane = 2% *w*/*w*; and cyclopentane = 2%, 4% and 6% *w*/*w*, but 6% was not suitable for use. These foam reactions were performed using the same methodology described for the preliminary tests (Section 2.3.1). These formulations are summarized in Table 1.

### 2.4. Characterization

#### 2.4.1. FTIR Analyses

Fourier transform infrared (FTIR) spectra were recorded on an ABB Bomer spectrometer using 0.1% KBr pellets and an ATR accessory with resolution of 4 cm^−1^, in the range of 4000–400 cm^−1^.

#### 2.4.2. Thermogravimetric Analyses (TGA and DTG)

The thermal behavior of the foams was evaluated by thermogravimetric analysis (TGA), which was carried out on a TA equipment model TA-50 Q at temperatures ranging from 30 °C to 800 °C with a heating rate of 10 °C·min^−1^ under a nitrogen flow of 40 mL·min^−1^.

#### 2.4.3. Morphology Analyses

The images obtained by optical microscopy were collected on an Olympus optical microscope, model BX41M, coupled to a TecVoz camera, model DNS 480. The morphology of polymers structure was characterized to observe the foam cellular structure by scanning electron microscope (SEM) images, which were obtained with a JSM–6360LV, a JEOL scanning microscope. 

#### 2.4.4. Determination of Apparent Density

Polyurethane foam densities were calculated according to the ASTM D-1622 standard by measuring three specimens of each sample with the dimensions of 50 × 50 × 25 mm. 

#### 2.4.5. Mechanical Properties

Mechanical testing (10% compressive strength) of polyurethane foams was performed on an Autograph Precision Universal Testing Machine AG-Xplus Series (Shimadzu, Kyoto, Japan), according to the ASTM D1621 standard method. At least five samples were analyzed to obtain the average value; the specimen size was 50 × 50 × 25 mm.

#### 2.4.6. Thermal Conductivity

Thermal conductivity tests were performed by an instrument developed by Professor Vagner Carvalho in the Surface Laboratory at the Physics Department at Universidade Federal de Minas Gerais—Brazil [29]. This equipment has a unit that contains a tip, which is electronically controlled and has a temperature sensor. The measurements are based on the thermal comparison method by copper-constantan differential thermocouples that measure the contact temperature gradient and the changes in the sample temperature. The samples were tested six times and a simple average was taken as the comparator output. A calibration curve was constructed using various ranges of samples with a wide thermal conductivity and the data were plotted in order to obtain the relation between the thermal conductivity and the comparator output.

## 3. Results and Discussion

### 3.1. Study of the Best Binary Mixture to Produce Polyurethane Foams

**Figure 1:**
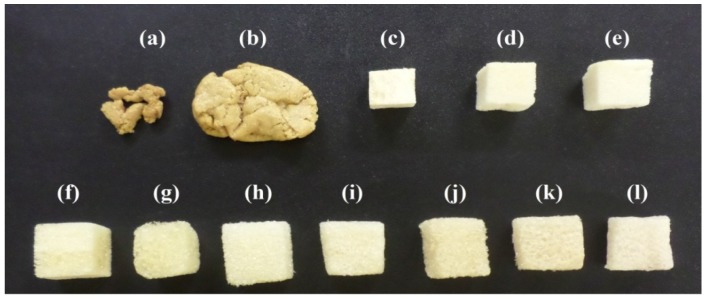
Foams produced varying the pure glycerol content: (**a**) 20%; (**b**) 40%; (**c**) 50%; (**d**) 60%; and (**e**) 80%,as well as varying the crude glycerol content: (**f**) 10%; (**g**) 20%; (**h**) 30%; (**i**) 40%; (**j**) 50%; (**k**) 60%; and (**l**) 70% of the polyols.

The binary polyol production was first studied by the physical mixture of pure glycerol and castor oil, varying the glycerol content. Some foams did not present good dimensional stability (Figure 1a). Upon increasing the content of pure glycerol, it was observed that the foams became denser and softer. The formulations with 20% and 40% (*w*/*w*) of pure glycerol content did not grow as a typical foam, yielding a very rigid, solid material. The foams produced with the polyol containing 50% (*w*/*w*) of pure glycerol presented a high homogeneity, but upon increasing this content, the foams became very friable.

The pure glycerol was then replaced by crude glycerol, a co-product of biodiesel production, in order to synthesize new foams and the results were quite different. The foams with crude glycerol and castor oil polyol (named GCo, Figure 1f–l) were more homogeneous and presented good dimensional stability in comparison to those synthesized with pure glycerol (Figure 1a–e). Based on these experimental behaviors, we believe that the crude glycerol impurities (alkaline catalyst, methanol, methyl esters of fatty acids, fatty acid methyl esters) are responsible for the best properties of the foams. Further investigations can be made to understand this behavior. Similar behaviors have already been reported in the literature, evaluating the effects of the replacement of pure glycerol by crude glycerol for producing polyols from biomass liquefaction. These studies also affirm that these impurities of crude glycerol improved the polyols’ and polyurethanes’ properties [9,11,30].

It was observed that upon increasing the amount of crude glycerol, there was a decrease in the rigidity and the dimensional stability of the foams. For this reason, the foam produced from the polyol containing 10% of crude glycerol and 90%of castor oil (*w*/*w*) (Figure 1f) was chosen to perform further studies. The hydroxyl number (240 mg·KOH·g^−1^) and viscosity (436.5 mm^2^·s^−1^) of this polyol were measured, indicating that these polyols are adequate to produce rigid foams [4]. Similar results have already reported in the literature for polyols from castor oil [26].

**Figure 2:**
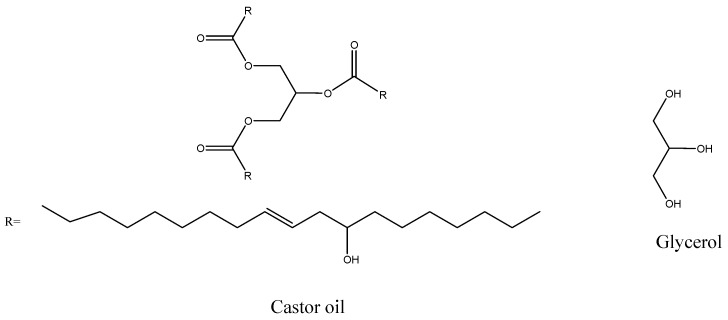
Structure of the castor oil (ricinoleic acid is the main component) and glycerol molecules.

It is important to point out that the polyol used to produce our best foam, with 10% of glycerol and 90% of castor oil (*w*/*w*), has the molar ratio glycerol/castor oil approximately equal to 1 (considering the molar weight of glycerol and castor oil 92.09 and 895.33 g·mol^−1^, respectively). Observing the structure of these molecules (Figure 2), there are three hydroxyl groups in each glycerol molecule and three instaurations of ricinoleic acid in the triglyceride structure that are suitable to be transformed into OH groups by pre-treatment reactions. Thus, we can consider that 1 mol of glycerol has the same number of OH groups as 1 mol of castor oil. Then, when we used a binary mixture 1:1, the OH amount is doubled. The same value of hydroxyl groups can be obtained by inserting an OH on each instauration of the castor oil ricinoleic chain. Our study was then performed using the binary mixture, without castor oil modification, to avoid extra costs in the process.

### 3.2. Study of Catalyst and Blowing Agent Effect in the Foams Properties

The characterization data obtained for the different foams, which were prepared using the best binary polyol (10% crude glycerol and 90% castor oil *w*/*w*) will be discussed in this section. The formulations will be presented using Roman numerals, as shown in Table 1.

**Figure 3:**
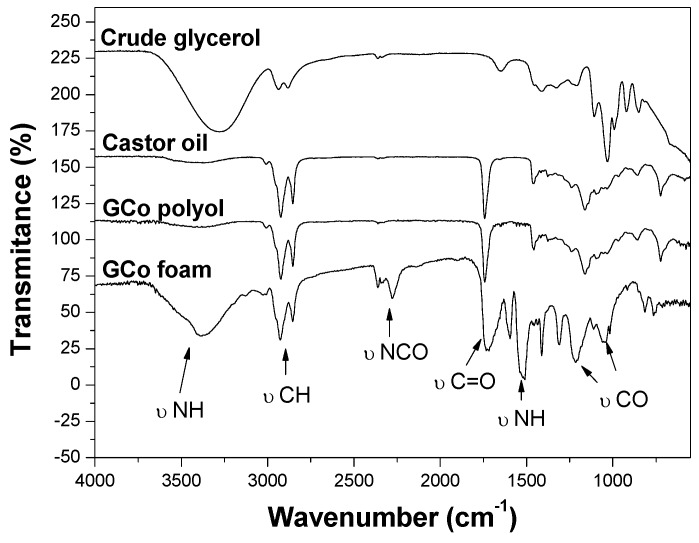
FTIR spectra for the raw materials, GCo polyol, and GCo foam (formulation II of Table 1)

The FTIR spectra of the renewable raw materials used to produce GCo polyols are shown in Figure 3. The band corresponding to the hydroxyl group’s vibration is observed at approximately 3700–3000 cm^−1^. The characteristic stretches of double bonds in the castor oil groups C=C–H and C=C are observed at 3020 and 1740 cm^−1^, respectively. The bands around 3018 and 2710 cm^−1^ are assigned to CH_2_ and CH_3_ stretches of aliphatic chains, which are quite pronounced in castor oil due to the 18-carbon chain. The characteristic band of carbonyl and carboxyl groups is observed to be centered at 1743 cm^−1^ in the castor oil spectrum. The alkenes deformation of CH_2_ groups, present in the castor oil structure, is observed in a strong band at 1458 cm^−1^. The bands around 1112–1000 cm^−1^ indicate the presence of primary and secondary hydroxyl groups. These bands are very pronounced in crude glycerol spectrum, due to the three hydroxyl groups present in its short chain [16,18].

All of the foams’ spectra produced from GCo polyols are very similar, while a typical foam spectrum is shown in Figure 3, which presents the characteristic polyurethane bands. The stretching and vibrations of NH groups were observed between 3808–3308, and 1512 and 1510 cm^−1^, respectively. The deformation of CH_2_ bonds was observed by the two thin bands at 2900 and 2890 cm^−1^. The vibration of N=C=N and N=C=O groups are attributed to bands between 2390 and 2150 cm^−1^. Other vibration modes of the CH bond were also observed at 1464, 1418, 1364, and 1294 cm^−1^. The band between 1730 and 1720 cm^−1^ corresponds to the stretch of the CO-free urethane bond, and around 1700 cm^−1^, the hydrogen bond between the carbonyl and hydrogen atoms (from NH groups) from urethane is also observed. A band related to stretching asymmetric links OCONH was revealed at 1380 cm^−1^. The bands between 1100 and 1000 cm^−1^ were attributed to primary and secondary hydroxyl groups [16,17].

**Figure 4:**
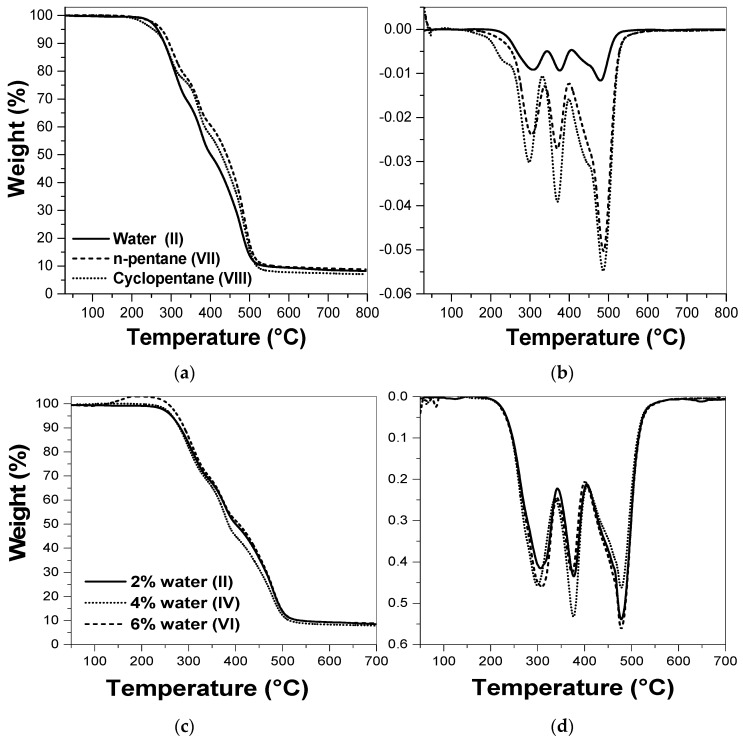
Thermogravimetric analysis: TGA (**a**,**c**) and DTG (**b**,**d**) curves of foams with GCo polyol with different types and amounts of blowing agents. (**a**,**b**) formulations II, VII, VIII; (**c**,**d**) formulations II, IV, VI of foams shown in Table 1.

The thermal behavior of the GCo foams containing different types and amounts of catalyst, shown in Figure 4, were evaluated by thermogravimetric analysis (TGA and DTG). The different foams presented similar thermal stability, and the DTG curves showed three regions of weight loss. The first event (around 300 °C) corresponds to urethane thermal degradation, free isocyanate, and alcohols; the second event is related to the degradation of rigid segments, at 370 °C; and the third event, approximately at 480 °C, is associated to the thermal degradation of the flexible segments and others segments of the remaining structure [31,32].

The effect of different blowing agents on the thermal stability of the GCo foams was evaluated, as shown in Figure 4a,b. The results indicate that the blowing agent type did not significantly modify the thermal behavior of the foams, which is suggested by the similar curves of the foams synthesized with water, cyclopentane, and n-pentane.

The effect of the amount of blowing agent (water) in the formulations (Figure 4c,d) was also investigated. The results show that the amount of water as a blowing agent did not significantly affect the thermal stability of the foams produced with polyol GCo, taking into account that all of the curves presented the same profile, indicating a similar thermal stability.

Apparent density is an important parameter of cellular polymers. The effect of the blowing agent type on the apparent density of the foams produced from GCo polyols (Table 2) showed that the formulations with physical blowing agents (cyclopentane and n-pentane) produced foams with higher densities than those synthesized with the chemical blowing agent (water). Similar results have been reported in the literature [32,33,34], and this behaviour indicates that smaller cells are formed due to the rapid volatilization of physical blowing agents, which have a low boiling point, during the highly exothermic foam growth step in comparison with the CO_2_, produced by the reaction of water with isocyanate [35].

**Figure 5:**
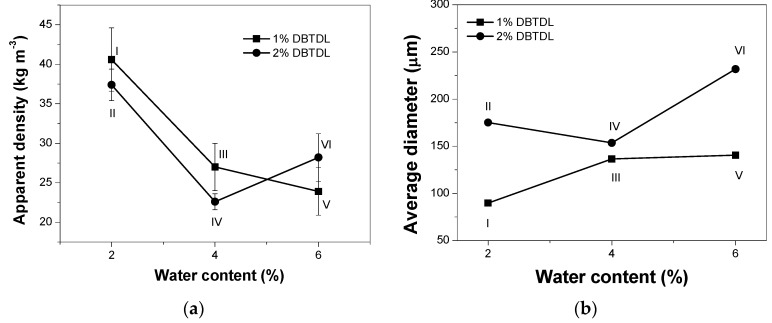
(**a**) Apparent density and (**b**) the average diameter of foams with different contents of the blowing agent (water) and catalyst. The numbers corresponding to the foam formulations (Table 1) are indicated in each point of these graphics.

The effect of the blowing agent (water) content on the foams’ apparent densities was also evaluated, as shown in Figure 5a. In increasing the water amount, a decrease of density is observed, which suggests that higher cells are formed with the enhancement of CO_2_ production from the water and isocyanate reaction [36]. 

Figure 5a also shows the influence of the catalyst content on the foams density. A decrease of apparent density was observed upon increasing the catalyst amount in the formulations. This behavior can be explained by the increment in the polymerization rate with the enhancement of the organometallic catalyst content in the formulation, avoiding the CO_2_ releasing during the foam’s cell formation [4]. As the reaction occurs with higher speed, the blowing agent is trapped in the structure and the cells presented higher diameters and lower densities (Figure 5a,b, respectively) [37]. This effect is more prominent in foams with higher water contents. These apparent density results are in agreement of the values measured for the same rigid polyurethane foams synthesized using castor oil polyols [19,26].

**Figure 6:**
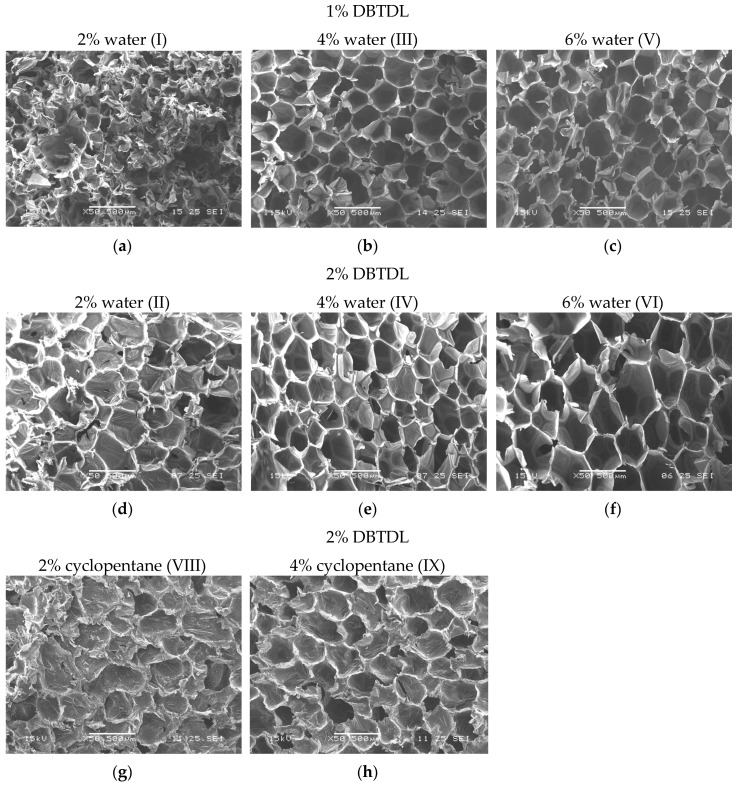
SEM micrographs of GCo foams with different types and contents of blowing agents and catalyst DBTDL (scale bar of 500 µm 50×). The numbers of the foam formulations (Table 1) are indicated in each micrograph.

The effect of different blowing agents on the cellular foam structures can also be observed in Figure 6, which shows SEM images of the foams synthesized with water and cyclopentane. The foams prepared with water as the blowing agent showed the largest cell size, confirming the density data (Figure 5a). Pentane has a low boiling temperature (around 50 °C) and volatizes very quickly, as previously explained in the density data discussion. The foam with 6% cyclopentane presented a low dimensional stability and, for this reason, its SEM micrograph was not shown here.

The foams formulated with water as the blowing agent presented the best dimensional stability, the lowest apparent density, and the higher cells homogeneity. Based on these results, we chose this formulation, aiming to evaluate the effect of the catalyst amount on the mechanical and conductivity properties. Another important aspect to note is that the use of water as a blowing agent is considered a green and inexpensive option.

The effect of the water content as the blowing agent was also evaluated by SEM images, as shown in Figure 6. It was observed that the water concentration is directly proportional to the cell size (Figure 5b). These analyses are in agreement with the density data (Figure 5a). The foams produced with 4% water presented higher cell homogeneity in comparison to those containing 2% water. The foams formulated with 6% water produced larger and heterogeneous cells, indicating that 4% water is the optimal quantity to be used in the foam formulations.

The comparison of the catalyst amount in the foam cells (Figure 6) formulated with water showed that an increment of the catalyst content yielded cellular materials with higher average cell diameters, confirming the density values in Figure 5a. The foams synthesized with 2% DBTDL showed the best cell homogeneity, despite higher cell diameters, as observed in Figure 5b. The average diameter of the foams produced in this present process is smaller than the data reported in the literature (from 107 to 121 µm) of foams synthesized from pre-polymerized castor oil [28], which is an important result for our foam uses. 

**Figure 7:**
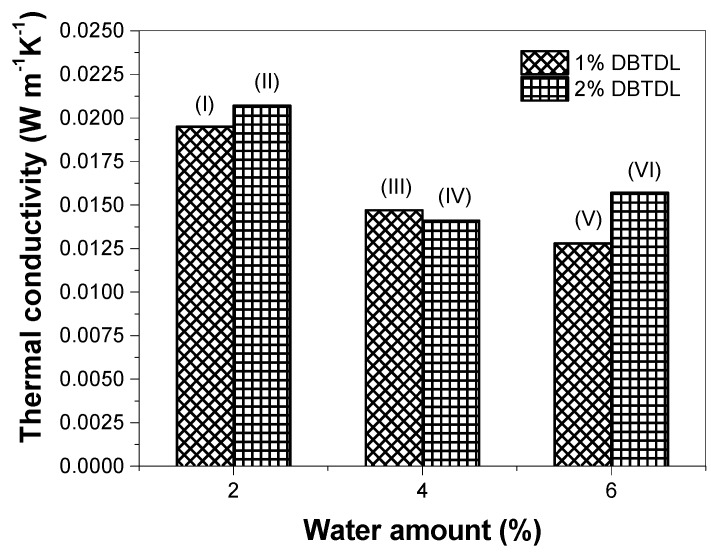
Thermal conductivity of foams with different contents of blowing agent (water) and catalyst (DBTDL). The numbers of foam formulations (Table 1) are indicated in each bar of this graphic.

The main property for the application of foam as thermal insulation is its thermal conductivity. This parameter was measured for the rigid foams synthesized with water as a blowing agent, and the results are presented in Figure 7. It was observed that upon increasing the water amount in these formulations, there was a decrease in the thermal conductivity. This result can be explained by the decreasing density and the enhancement of the average cell diameter of the foams [38]. 

The effect of the catalyst amount on this property is also presented in Figure 7. The use of a higher catalyst content in the formulations causes a slight thermal conductivity value enhancement, despite the density decrease as a consequence of cell size increase, as shown in Figure 5. The foams synthesized in this study presented better results in comparison to those reported in the literature for foams derived from renewable raw materials, whose values vary between 0.0233 and 0.0505 W·m^−1^·K^−1^, suggesting that these materials have a potential use as thermal insulation [22,39,40]. These thermal conductivity results are also better than those found for foams produced from pre-treated castor oil, especially if we consider the use of the very simple and inexpensive method of production [19,28].

**Figure 8:**
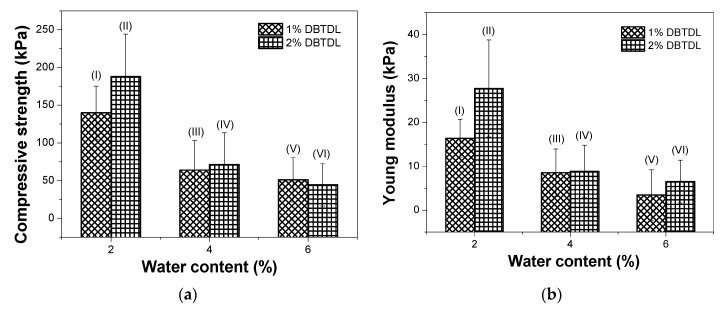
(**a**) Compressive strength and (**b**) Young’s modulus of foams with different contents of blowing agent (water) and catalyst (DBTDL). The numbers corresponding to the foam formulations (Table 1) are indicated in each point of these graphics.

The mechanical properties of the foams synthesized with different contents of blowing agent and catalyst were evaluated, and the results are shown in Figure 8. These results presented similar values to those reported in the literature of foams produced from castor oil polyols, which ranged from 125 to 220 kPa [16,19,25,26]. A significant decrease in the compressive strength and Young’s modulus of the foams were observed with the addition of higher blowing agent amounts, which can be related with the decrease in density and the increase in cell size. As the cell structure becomes higher, less force is necessary to cause deformation in these foams [36].

The results of the compressive strength and Young’s modulus of foams with different amounts of catalyst in the formulations (Figure 8a,b) showed that there are no significant changes in the values upon increasing the catalyst amount, especially for the formulations with 4% and 6% of water as the blowing agent. The variations are within the experimental errors.

Comparing all of the formulations, it was observed that the foam with best thermal conductivity (0.0141 W·m^−1^·K^−1^) was formulated with 1% of DBTDL and 6% of water, which also showed a low apparent density value (23.9 kg·m^−3^). However, this sample showed a low compressive strength (51.01 kPa) and Young’s modulus (3.44 kPa), suggesting its application as an insulator of places that do not receive high loads. The foam containing 2% of DBTDL and 2% of water possess a higher compressive strength (187.93 kPa) and Young’s modulus (27.74 kPa), as well as a low apparent density value (37.4 kg·m^−3^). On the other hand, the thermal conductivity value was the higher (0.0207 W·m^−1^·K^−1^) in comparison with the other formulations; indeed, this insulation property value is in the range of typical commercial products [2].

## 4. Conclusions

Rigid polyurethane foams were synthesized from a polyol produced by the physical mixture of castor oil and crude glycerol, a co-product from the biodiesel industry. The polyol preparation method is simple and does not require any pre-treatment of the raw materials. The best formulation was obtained with 10% of crude glycerol and 90% of castor oil (*w*/*w*, molar ratio = 1:1) and water as the blowing agent. The addition of 10% glycerol increased the OH content of the biopolyol to the same extent as if the castor oil had been pre-treated in order to insert one hydroxyl group in each instauration (C=C) of ricinoleic acid.

The effect of the blowing agent on the foam synthesis was evaluated and the results showed that increasing the water amount caused a decrease in density, thermal conductivity, compressive strength, and Young’s modulus. These behaviors are due to the cell size enhancement.

The evaluation of the effect of the catalyst (DBTDL) amount showed that increasing the catalyst content caused a decrease in density and an increase in thermal conductivity, however, there was only a slight influence on the compressive strength and Young’s modulus. 

These innovative foams presented properties that indicate their great potential to be used as thermal insulation. These materials are inexpensive, environmentally friendly, and can contribute to reducing the biodiesel prices in a biorefinery approach. These biopolymers can be synthesized by a simple process, which can be easily used in a large scale. 

## Figures and Tables

**Table 1:** Formulations of the foams prepared with GCo polyol (10% crude glycerol and 90% castor oil *w*/*w)*, NCO/OH = 2.0, and 2% Tegostab (surfactant).

Formulations	Blowing Agent (Type)	Blowing Agent (%)	Catalyst DBTDL (%)
I	Water	2	1
II	Water	2	2
III	Water	4	1
IV	Water	4	2
V	Water	6	1
VI	Water	6	2
VII	n-pentane	2	2
VIII	ciclopentane	2	2
IX	ciclopentane	4	2

**Table 2:** Density values of foams with different blowing agents.

Formulation	Blowing Agent	Apparent Density (kg·m^−3^)
II	Water	37.4
VII	n-pentane	61.3
VIII	Cyclopentane	99.3

## References

[1] Grand View Research Global Rigid Polyurethane (PU) Foams Industry Trends And Market Segment Forecasts To 2020–Worldwide Rigid Polyurethane (PU) Foams Market, Product (Molded Foam Parts, Slabstock Polyether, Slabstock Polyester), and Segment Forecast to 2020. http://www.grandviewresearch.com/industry-analysis/rigid-polyurethane-pu-foams-industry.

[2] Zhang H., Fang W.-Z., Li Y.-M., Tao W.-Q. (2017). Experimental study of the thermal conductivity of polyurethane foams. Appl. Therm. Eng..

[3] Tibério Cardoso G., Claro Neto S., Vecchia F. (2012). Rigid foam polyurethane (PU) derived from castor oil (*Ricinus communis*) for thermal insulation in roof systems. Front. Archit. Res..

[4] Vilar W. (2004). Química e Tecnologia dos Poliuretanos.

[5] Xue B.L., Wen J.L., Sun R.C. (2014). Lignin-based rigid polyurethane foam reinforced with pulp fiber: Synthesis and characterization. ACS Sustain. Chem. Eng..

[6] Cinelli P., Anguillesi I., Lazzeri A. (2013). Green synthesis of flexible polyurethane foams from liquefied lignin. Eur. Polym. J..

[7] Araújo R.C.S., Pasa V.M.D., Melo B.N. (2005). Effects of biopitch on the properties of flexible polyurethane foams. Eur. Polym. J..

[8] Melo B.N., Pasa V.M.D. (2004). Thermal and morphological study of polyurethanes based on eucalyptus tar pitch and castor oil. J. Appl. Polym. Sci..

[9] Hu S., Li Y. (2014). Polyols and polyurethane foams from acid-catalyzed biomass liquefaction by crude glycerol: Effects of crude glycerol impurities. J. Appl. Polym. Sci..

[10] Hu S., Li Y. (2014). Two-step sequential liquefaction of lignocellulosic biomass by crude glycerol for the production of polyols and polyurethane foams. Bioresour. Technol..

[11] Hu S., Wan C., Li Y. (2012). Production and characterization of biopolyols and polyurethane foams from crude glycerol based liquefaction of soybean straw. Bioresour. Technol..

[12] Narine S.S., Kong X., Bouzidi L., Sporns P. (2007). Physical properties of polyurethanes produced from polyols from seed oils: I. Elastomers. J. Am. Oil Chem. Soc..

[13] Hatakeyama H., Matsumura H., Hatakeyama T. (2013). Glass transition and thermal degradation of rigid polyurethane foams derived from castor oil-molasses polyols. J. Therm. Anal. Calorim..

[14] Cangemi J.M., dos Santos A.M., Neto S.C., Chierice G.O. (2008). Biodegradation of polyurethane derived from castor oil. Polímeros.

[15] Chethana M., Madhukar B.S., Siddaramaiah, Somashekar R. (2014). Structure-property relationship of biobased polyurethanes obtained from mixture of naturally occurring vegetable oils. Adv. Polym. Technol..

[16] Li Q.F., Feng Y.L., Wang J.W., Yin N., Zhao Y.H., Kang M.Q., Wang X.W. (2016). Preparation and properties of rigid polyurethane foam based on modified castor oil. Plast. Rubber Compos..

[17] Ristić I.S., Bjelović Z.D., Holló B., Mészáros Szécsényi K., Budinski-Simendić J., Lazić N., Kićanović M. (2013). Thermal stability of polyurethane materials based on castor oil as polyol component. J. Therm. Anal. Calorim..

[18] Zhang L., Zhang M., Hu L., Zhou Y. (2014). Synthesis of rigid polyurethane foams with castor oil-based flame retardant polyols. Ind. Crops Prod..

[19] Zhang M., Pan H., Zhang L., Hu L., Zhou Y. (2014). Study of the mechanical, thermal properties and flame retardancy of rigid polyurethane foams prepared from modified castor-oil-based polyols. Ind. Crops Prod..

[20] Badri K.H. (2012). Biobased polyurethane from palm kernel oil-based polyol: Polyurethane. Polyurethane.

[21] Chuayjuljit S., Maungchareon A., Saravari O. (2010). Preparation and Properties of Palm Oil-Based Rigid Polyurethane Nanocomposite Foams. J. Reinf. Plast. Compos..

[22] Tan S., Abraham T., Ference D., MacOsko C.W. (2011). Rigid polyurethane foams from a soybean oil-based Polyol. Polymer.

[23] Yoshioka M., Nishio Y., Saito D., Ohashi H., Hashimoto M., Shiraishi N. (2013). Synthesis of biopolyols by mild oxypropylation of liquefied starch and its application to polyurethane rigid foams. J. Appl. Polym. Sci..

[24] Kim D., Kwon O., Yang S., Park J., Chun B.C. (2007). Structural, Thermal, and Mechanical Properties of Polyurethane Foams Prepared with Starch as the Main Component of Polyols. Fibers Polym..

[25] Veronese V.B., Menger R.K., Madalena M., Forte D.C., Petzhold C.L. (2011). Rigid Polyurethane Foam Based on Modified Vegetable Oil. J. Appl. Polym. Sci..

[26] Ionescu M., Radojči D., Wan X., Shrestha M.L., Petrovi Z.S., Upshaw T.A. (2016). Highly functional polyols from castor oil for rigid polyurethanes. Eur. Polym. J..

[27] Stirna U., Lazdina B., Vilsone D., Lopez M.J., Vargas-Garcia M.D.C., Suarez-Estrella F., Moreno J. (2012). Structure and properties of the polyurethane and polyurethane foam synthesized from castor oil polyols. J. Cell. Plast..

[28] Hejna A., Kirpluks M., Kosmela P., Cabulis U., Haponiuk J., Piszczyk L. (2017). The influence of crude glycerol and castor oil-based polyol on the structure and performance of rigid polyurethane-polyisocyanurate foams. Ind. Crops Prod..

[29] Carvalho V.E. (1978). Contrução de um Comparador Térmico de Leitura Direta.

[30] Hu S., Li Y. (2014). Polyols and polyurethane foams from base-catalyzed liquefaction of lignocellulosic biomass by crude glycerol: Effects of crude glycerol impurities. Ind. Crop. Prod..

[31] Corcuera M.A., Rueda L., Fernandez d’Arlas B., Arbelaiz A., Marieta C., Mondragon I., Eceiza A. (2010). Microstructure and properties of polyurethanes derived from castor oil. Polym. Degrad. Stab..

[32] Carriço C.S., Fraga T., Pasa V.M.D. (2016). Production and characterization of polyurethane foams from a simple mixture of castor oil, crude glycerol and untreated lignin as bio-based polyols. Eur. Polym. J..

[33] Modesti M., Adriani V., Simioni F. (2000). Chemical and physical blowing agents in structural polyurethane foams: Simulation and characterization. Polym. Eng. Sci..

[34] Lim H., Kim E.Y., Kim B.K. (2010). Polyurethane foams blown with various types of environmentally friendly blowing agents. Plast. Rubber Compos..

[35] Choe K.H., Lee D.S., Seo W.J., Kim W.N. (2004). Properties of Rigid Polyurethane Foams with Blowing Agents and Catalysts. Polym. J..

[36] Thirumal M., Khastgir D., Singha N.K., Manjunath B.S., Naik Y.P. (2008). Effect of Foam Density on the Properties of Water Blown Rigid Polyurethane Foam. J. Appl. Polym. Sci..

[37] Mills N.J. (2007). Polymer Foams Handbook : Engineering and Biomechanics Applications and Design Guide.

[38] Jarfelt U., Ramnas O. Thermal conductivity of polyurethane foam–best performance. Proceedings of the 10th International Symposium on District Heating and Cooling.

[39] Ribeiro Da Silva V., Mosiewicki M.A., Yoshida M.I., Coelho Da Silva M., Stefani P.M., Marcovich N.E. (2013). Polyurethane foams based on modified tung oil and reinforced with rice husk ash I: Synthesis and physical chemical characterization. Polym. Test..

[40] Gama N.V., Soares B., Freire C.S.R., Silva R., Neto C.P., Barros-Timmons A., Ferreira A. (2015). Bio-based polyurethane foams toward applications beyond thermal insulation. Mater. Des..

